# The taste of togetherness

**DOI:** 10.7554/eLife.05490

**Published:** 2014-12-11

**Authors:** Jonathan Trevorrow Clark, Anandasankar Ray

**Affiliations:** Interdisciplinary Neuroscience Program, University of California Riverside, California, United States; Entomology Department, the Institute for Integrative Genome Biology and the Interdisciplinary Neuroscience Program, University of California Riverside, California, United States, anand.ray@ucr.edu

**Keywords:** Drosophila melanogaster, Drosophila sechellia, Drosophila simulans, pheromones, pickpocket, social behavior, *D. melanogaster*

## Abstract

The larvae of fruit flies produce pheromones to control whether they are attracted to others of the same species or whether they avoid members of a different species.

**Related research article** Mast JD, De Moraes CM, Alborn HT, Lavis LD, Stern DL. 2014. Evolved differences in larval social behavior mediated by novel pheromones. *eLife* **3**:e04205. doi: 10.7554/eLife.04205

**Image** The brain region of a fruit fly larva where the pheromone-sensing neurons expressing the ion channel *pickpocket29* (green) connect

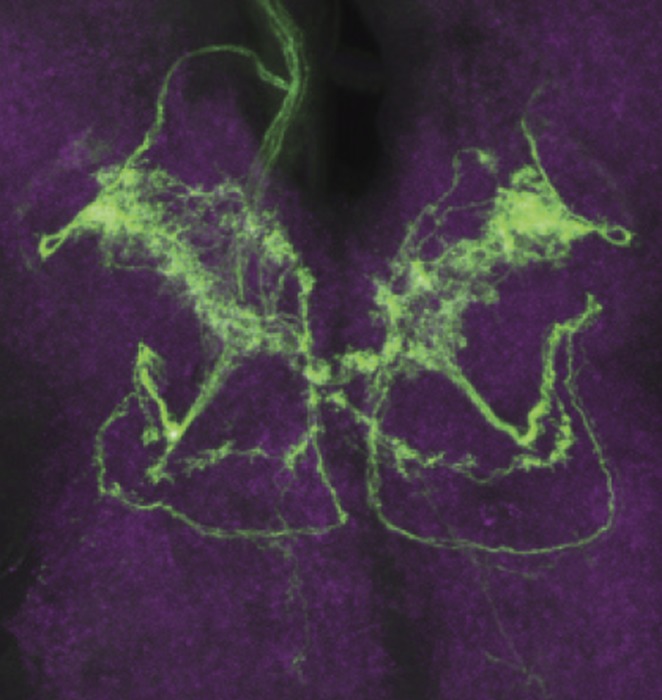

A wide range of organisms, including insects, can communicate with other organisms of the same species using sounds, signs and chemicals. The chemical cues emitted by organisms are called pheromones, and they trigger important behaviors like courtship, aggression, aggregation and aversion. The behavior produced by a pheromone depends on the chemical released and the social context in which it is detected.

Understanding how pheromones drive such powerful behaviors can be studied in depth in the fruit fly *Drosophila melanogaster* because its relatively simple nervous system makes it easy to investigate the neural circuits that detect and respond to these chemical signals. Several important principles have emerged from extensive studies of pheromone signaling in adult fruit flies, including an understanding of how pheromones are detected and how they can influence behavior in male and female flies in different ways. However, most of this work has focused on a pheromone called cis-vaccenyl acetate (van der Goes van Naters and Carlson, 2007). Now in *eLife,* Joshua Mast and colleagues from the Janelia Farm Research Campus, ETH Zürich and the US Department of Agriculture have used the chemosensory system of fruit fly larvae to identify new pheromones and the neurons that detect them (Mast et al., 2014).

*D. melanogaster* larvae have chemosensory (chemical detection) systems that are much simpler than those found in adults. This, along with the simple behavior of the larvae, makes them very suitable for studying pheromones. *D. melanogaster* larvae gather together on food sources, suggesting that they may produce pheromones that cause them to aggregate (Durisko et al., 2014). However, the identity of these pheromones remained a mystery.

Mast and co-workers now show that *D. melanogaster* larvae are attracted to surfaces on which other larvae of the same species have crawled, and have found that this behavior is caused by a chemical cue deposited by the larvae. Detecting this cue requires two ion channels, called *pickpocket23* and *pickpocket29,* that are expressed in the neurons of the terminal organ, which is the main taste organ of a larva (Figure 1). Taste neurons expressing these two ion channels are also known to detect sex pheromones in adult *D. melanogaster* flies (Lu et al., 2012; Thistle et al., 2012; Toda et al., 2012). The simplicity of the larval neural circuits allowed Mast et al. to narrow down the possible neurons that could detect pheromones to a single pair of neurons (R58F10+) in the terminal organ. This finding also provides a foundation for future investigations into the precise regions of the brain to which this pair of neurons sends information.

**Figure 1.**
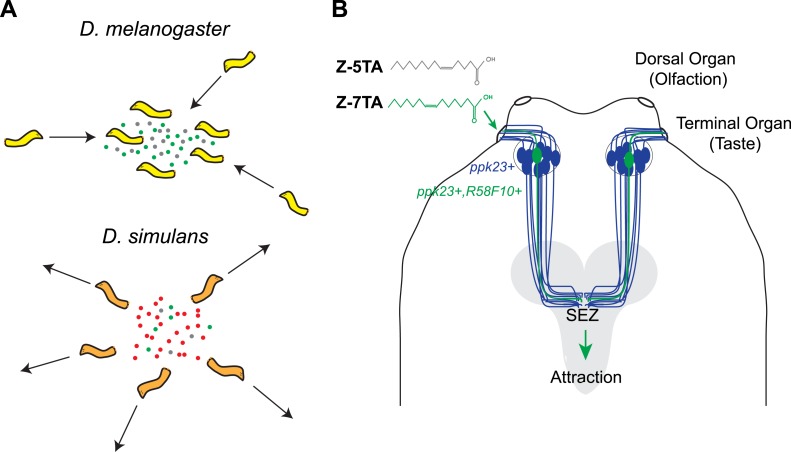
Different species of fruit fly larvae use pheromones to either find or avoid one another. (**A**) Mast et al. reveal that the attractive pheromones (Z)-5-tetradecenoic acid (Z-5-TA; gray dots) and (Z)-7-tetradecenoic acid (Z-7-TA; green dots) are secreted by the larvae of *D. melanogaster* and cause other larvae to gather together (top). The larvae of *D. simulans* produce these pheromones at lower levels, and are thought to produce an additional compound (red dots) that repels other larvae (bottom). (**B**) Dorsal view of the ‘head’ of a *D. melanogaster* larva. A single pair (green) of the 10 pairs of *pickpocket23*-expressing neurons (blue) extends into the terminal organ and detects Z-5-TA and Z-7-TA. This information is then relayed to a collection of neurons in the subesophageal zone (SEZ) of the brain (grey).

Next, Mast et al. used gas chromatography to separate the attractive chemicals deposited by the larvae, and then used mass spectrometry to identify a small number of compounds. Examining how the larvae responded to synthetic versions of each compound identified two rare fatty acids—(Z)-5-tetradecenoic acid and (Z)-7-tetradecenoic acid—as being responsible for attracting the larvae to each other. The pair of *pickpocket23*-expressing neurons in the terminal organ is able to detect both of these chemicals.

During courtship, pheromones are often used to signal that both participants are of the same species (Savarit et al., 1999; Ferveur, 2005). Generally, members of different *Drosophila* species will not court flies from other species because, it is thought, each species has a characteristic pheromone profile (Coyne et al., 1994). This species-specific effect is also partially seen in response to the pheromones that cause the larvae to aggregate: *D. melanogaster* larvae will gather together to form aggregates, but the larvae of a closely related species, *Drosophila simulans*, will not (Durisko et al., 2014).

Mast et al. reasoned that these differences in behavior could have several underlying causes. *D. simulans* may have lost sensitivity to the pheromones that *D. melanogaster* larvae find attractive, or may produce different pheromones—or a combination of these possibilities. Tests revealed that the lack of aggregation in *D. simulans* larvae is likely the result of the reduced production of both of the attractive *D. melanogaster* pheromones detected by Mast et al., as well as the production of an additional unknown repellant compound.

The differences in pheromone signaling between species are of great interest in evolutionary biology, as they could be key for establishing new species by preventing separate species breeding with each other (Cobb and Hallon, 1990; Ferveur, 2010). The demonstration by Mast et al. that the larvae of different, closely related, fly species produce different pheromones suggests that pheromones are able to influence social behavior even at that early developmental stage. Exactly how these pheromones evolved along with their receptor systems and circuits, and how they participate in species-specific adaptations, remain open questions for future investigation.
